# Editorial: Translational Side of Emerging Invasive and Non-invasive Stimulation Therapies

**DOI:** 10.3389/fnins.2022.872551

**Published:** 2022-03-23

**Authors:** Leo K. Cheng, Luming Li, Bruno Bonaz, Jiande D. Z. Chen

**Affiliations:** ^1^Auckland Bioengineering Institute, University of Auckland, Auckland, New Zealand; ^2^National Engineering Laboratory for Neuromodulation, School of Aerospace Engineering, Tsinghua University, Beijing, China; ^3^Centre Hospitalier Universitaire Grenoble Alpes, Grenoble, France; ^4^Division of Gastroenterology and Hepatology, University of Michigan, Ann Arbor, MI, United States

**Keywords:** deep brain stimulation (DBS), vagal nerve stimulation (VNS), sacral nerve stimulation (SNS), gastric electrical stimulation, transcutaneous auricular vagus nerve stimulation (taVNS), ultrasound stimulation, electromagnetic field stimulation, sciatic nerve stimulation

In the past decades, neuroscience research has witnessed dramatic progress in the field of neuromodulation, a stimulation technology that delivers electrical stimuli to modulate nerve activity in targeted areas or organs. It is widely used for the treatment of chronic pain (Coffey, 2001; Ostergard et al., 2014), and also used to modulate the function of target organs such as the heart, gastrointestinal tract or bladder (van Balken et al., 2004; Yin and Chen, 2008; Payne et al., 2019). It is a non-drug-based therapy, generally safe and, in particular, without the side effects of pharmacological treatments.

This Frontiers Research Topic on *Translational Side of Emerging Invasive and Non-invasive Stimulation Therapies* was instigated, in part, due to presentations and discussions at the International Neuromodulation Society's 14th World Congress held in Sydney, Australia, on 25–30 May 2019. The topic editors for this Frontiers Research topic were Professors Jiande Chen, Bruno Bonaz, Leo Cheng, and Luming Li. In total, 93 authors contributed to 14 articles that were published in Frontiers in Neuroscience, Frontiers in Neurology and Frontiers in Physiology.

Functional gastrointestinal disorders are widespread, impart significant economic burden and can greatly reduce quality of life of patients (Everhart and Ruhl, 2009; Sperber et al., 2020). Disorders of the stomach include gastroparesis, functional dyspepsia. Only one device is currently available for delivering gastric electrical stimulation and used for treating nausea and vomiting in patients with gastroparesis (McCallum et al., 2011). However, its efficacy and mechanisms of action remain uncertain (Ducrotte et al., 2020). Cheng et al. review the large variety of electrical stimulation and pacing parameters applied directly to the stomach musculature. Strategies are described to optimize and standardize these stimulation parameters as well as techniques to investigate the mechanisms underlying stimulation techniques. However, it is clear that a number of key challenges remain before such methods are widely used for treating functional gastrointestinal disorders. These include the development of methods to reliably quantify the functional responses to electrical therapies, and the convergence of pacing and stimulation protocols that are able to sustain long-term responses. The integration of mathematical modeling techniques with clinical trials will help to refine and accelerate the development of stimulation protocols and devices. Vagal nerve stimulation is another key target and has been introduced to treat inflammatory bowel diseases such as Crohn's disease and ulcerative colitis (Bonaz et al.). Bonaz et al. review the anti-inflammatory actions of vagal nerve stimulation. Inflammatory bowel disease is a major healthcare concern worldwide, especially in developed countries: it is of a high prevalence, no medications to cure and expensive and difficult to treat. Vagal nerve stimulation has a great potential for treating inflammatory bowel disease mediated via the cholinergic anti-inflammatory pathway. In the review, Bonaz et al. provides neuroanatomical basis of vagal nerve stimulation and discusses how vagal nerve stimulation may improve abnormalities in brain-gut axis. Recently, sacral nerve stimulation was reported to exert similar anti-inflammatory effects in animal models of colitis (Guo et al., 2019) mediated via the spinal afferent and vagal efferent pathways (Tu et al., 2020). The sacral nerve is another branch of the parasympathetic nervous system which innervates the distal colon and rectum as well as other pelvic organs, such as the bladder and genitals. Sacral nerve stimulation is clinically approved for treating overactive bladder, urinary retention and fecal incontinence. In this Research Topic, Jin et al. investigated the use of sacral nerve stimulation for the treatment of visceral hypersensitivity underlying irritable bowel syndrome. In a rodent model of colonic hypersensitivity, sacral nerve stimulation improved visceral pain by inhibiting mast cell overactivation in the colon tissue via the modulation of the autonomic function. Functional anorectal pain is another disorder that is poorly understood. The pathophysiology of this disorder was investigated and a correlation between anal pressure and afferent signaling was determined in a topic article (Zhang et al.).

Deep brain stimulation is one of the most important clinical therapies for several neurological disorders, such as Parkinson's disease, movement disorders, major depression and addiction. Three of the articles in this Frontiers Topic focussed on deep brain stimulation. Sui et al. present a comprehensive review on technical challenges, clinical applications and perspectives and mechanisms of action. Accurate lead localization is an important issue in deep brain stimulation, He and colleagues presented an magnetic resonance-based assessment method to determine the lead localization deviation (He et al.). Appropriate patient screening is critically important in various medical therapies and this is also the case for deep brain stimulation. Wang et al. investigated the predictivity of deep brain stimulation outcome in Parkinson's diseases based on functional connectivity assessed from the rest-state functional magnetic resonance imaging.

A number of commercial devices have received Food and Drug Administration (FDA) approval for the treatment of lower urinary tract dysfunction (van Balken et al., 2004; Siddiqui et al., 2010). Payne et al. developed and applied a new electrode array to record and stimulate the pelvic nerves in conscious rats. They showed that the electrode array could be used for the development of closed-loop neuromodulation devices targeting urological dysfunction. To aid in the development of closed-loop devices, Chen Y. et al. described a method for filtering out electrocardiogram artifacts by sensing-enabled neurostimulator.

Non-invasive electrical stimulation methods are of great interest and provide an attractive option for treating disorders as they do not require surgical implantation of a stimulator or stimulation leads. Chen M. et al. reviewed non-invasive methods for treating cardiac disorders resulting from autonomic imbalance. Various transcutaneous approaches are discussed in this Research Topic, including, transcutaneous auricular vagus nerve stimulation (taVNS), ultrasound stimulation and electromagnetic field stimulation. Andersen et al. presented the use of peripheral electrical stimulation using a circular electrode array on the surface of the forearm with the aim of treating chronic pain (Arendsen et al.) while Machetanz et al. used taVNS to modulate heart function and brain activity.

Of current interest during the current COVID-19 pandemic, Lu et al. presented a technique for remote and wirelessly programming a sacral nerve stimulator for treating chronic pain. Such a method is a critical advance as the pandemic has resulted in major impact on the healthcare system and the ability for patients to travel to their local hospitals.

Diabetes affects 5–10% of general population and is a huge healthcare burden around the world. Type 2 diabetes, a major subtype of diabetes, is closely associated with obesity. Vagal nerve stimulation, a method called VBLOC, has received FDA approval for treating obesity although it has not been widely utilized due to its limited clinical efficacy (Apovian et al., 2017). Gastric electrical stimulation, intestinal electrical stimulation and vagal nerve stimulation have been explored for treating diabetes (Liu et al., 2011; Lebovitz, 2016; Yin et al., 2019; Dong et al., 2021); however, there is a lack of clinical efficacy data. In this Research Topic, Beeve et al. applied sciatic nerve stimulation and reported its neuroskeletal effects in an animal model of type 1 diabetes.

In summary, non-invasive methods may be appropriate for common but relatively mild or moderate disorders, while invasive therapies using an implantable device may be reserved for chronic and severe conditions. In general, the articles in this Frontiers Research Topic have indicated that significant research should be conducted to improve our understanding of the mechanisms underlying these complex disorders. A focus should also be placed on translating methods and findings from animal studies to patients.

## Author Contributions

LC wrote the first draft. All authors conceived the concept and revised the manuscript. All authors contributed to the article and approved the submitted version.

## Funding

This work was funded, in part, by the Marsden Fund Council managed by Royal Society Te Apārangi, Ministry of Business Innovation and Employment's Ministry of Business, Innovation and Employment's Catalyst: Strategic fund (for LC), and the National Institutes of Health (NIH) (R01 HD088662 and SPARC OT2OD030538 for LC; and R42DK100212 and 1UG3NS115108 for JC).

## Conflict of Interest

The authors declare that the research was conducted in the absence of any commercial or financial relationships that could be construed as a potential conflict of interest.

## Publisher's Note

All claims expressed in this article are solely those of the authors and do not necessarily represent those of their affiliated organizations, or those of the publisher, the editors and the reviewers. Any product that may be evaluated in this article, or claim that may be made by its manufacturer, is not guaranteed or endorsed by the publisher.
